# Silicon Carbide Neural Interfaces: A Review of Progress Toward Monolithic Devices

**DOI:** 10.3390/nano15241880

**Published:** 2025-12-15

**Authors:** Christopher L. Frewin, Matthew Melton, Evans Bernardin, Mohammad Beygi, Chenyin Feng, Stephen E. Saddow

**Affiliations:** 1 Crystal Cybernetics LLC, Monroe, MI 48161, USA; chrislfrewin@gmail.com; 2 Department of Medical Engineering, University of South Florida (USF), Tampa, FL 33620, USA; mmelton3@usf.edu; 3 Department of Electrical Engineering, University of South Florida (USF), Tampa, FL 33620, USA; ebernardin@usf.edu (E.B.);; 4 Department of Mechanical Engineering, University of South Florida (USF), Tampa, FL 33620, USA; fcy@hubu.edu.cn

**Keywords:** neural interfaces, silicon carbide, monolithic SiC technology

## Abstract

The promise of intracortical neural interfaces—to restore lost sensory and motor function and probe the brain’s activity—has long been constrained by device instability over chronic implantation. Conventional silicon-based probes, composed of heterogeneous materials, often fail due to mechanical mismatch, inflammatory responses, and interface-driven degradation, where stress can induce cracking, swelling, and exposure of cytotoxic elements to neural tissue. Silicon carbide (SiC) offers a compelling solution, combining chemical inertness, structural strength, and biocompatibility in both amorphous and crystalline forms. In this review, we discuss advances in SiC neural interfaces, highlighting contributions from multiple laboratories and emphasizing our own work on monolithic devices, constructed entirely from a single, homogeneous SiC material system. These devices mitigate interface-driven failures and show preliminary indications of magnetic resonance imaging (MRI) compatibility, with minimal image artifacts observed compared to conventional silicon probes, though further in vivo studies are needed to confirm thermal safety at high-field conditions. Collectively, SiC establishes a versatile platform for next-generation, durable neural interfaces capable of reliable, long-term brain interaction for both scientific and clinical applications.

## 1. Introduction

Interaction with neurons using electrical signals has been a reality since the 18th century; however, this interaction has mainly been limited to gross stimulation of neural regions with no respect to closed-loop operation or bidirectional activity. In the 20th century, improved manufacturing capabilities for microelectrodes led to the inception of the brain–computer interface (BCI), where computer or microcontroller chips could interact directly with neurons using bidirectional signals from electrodes to provide therapeutic relief for a multitude of neural issues and diseases [1,2]. The BCI now includes brain machine interfaces (BMI), which refers to a computer/neural interaction to control mechanical prosthetics [1]. More than 2 million people are living with limb loss in the United States alone, and this number is expected to nearly double (3.6 million) by 2050 [3]. Millions of additional individuals are affected by neural diseases like Parkinson’s and dementia, and many more experience the loss of sensory functions [4]. Stimulation using macroelectrodes, like deep brain stimulators, has provided some therapeutic relief for individuals with these issues, but the large size of the stimulators has demonstrated side effects that arise from the stimulation of non-targeted neural regions [5].

Microelectrodes consisting of micro-sized wires were developed in the late 1950s to be able to directly interact with individual neurons, allowing direct recording of their action potentials and limiting stimulation to very localized, small regions [6,7]. Improvements in silicon device manufacturing led to silicon-based probes, like the planar Michigan multielectrode probe and the bulk silicon Utah electrode [8,9]. While these microelectrode types have demonstrated excellent bidirectional signal communication when interacting with individual neurons, they have expressed unreliable chronic, long-term signal capability, which manifests in the loss of action potential recordings and the increase in stimulation requirements for these devices [10,11,12,13]. The loss of microelectrode device reliability has been attributed to a multitude of biotic and abiotic issues, with a direct focus on the materials of device fabrication. One major biotic issue is the foreign body response, which leads to tissue loss around the implant and eventual glial encapsulation of microelectrode devices [12,13,14,15]. Another biotic issue is the modular mismatch between the microelectrode devices and the soft neural tissue that has been attributed to additional tissue damage over time [15,16]. Many of the abiotic issues are linked to delamination, cracking, and swelling of the devices due to water uptake [17,18,19]. Microelectrodes are typically fabricated using heterogeneous materials, in other words, different materials for the substrate, insulation, and electrode. This adds multiple complications to chronic device interaction, as each material introduces its own biotic and abiotic issues to the overall reliability of the device and can negatively impact the long-term signal reliability of microelectrodes [20].

In 2016, Barrese et al. conducted a systematic study of the interaction between Utah-style intracortical arrays chronically implanted within non-human primate tissue [21]. This study was an excellent demonstration of the loss of microelectrode reliability due to the activity of heterogeneous material interactions. The microelectrode devices demonstrated glial inflammatory reactions similar to multiple studies, and the observed inflammation stabilized within 6 to 12 weeks post-implantation. One additional biotic concern arose from increased encapsulation from meningeal cells traveling down the device to restore the dural barrier. The study attributed much of the device failure to the delamination and cracking of the parylene-C insulation encapsulation, which was attributed to long-term cerebral spinal fluid exposure increasing the crystallinity and brittleness of the material. Increased delamination was observed to be due to cellular invasion into the resulting cracks. The platinum electrodes demonstrated increased corrosion over time due to slow oxidation from the inflammatory response. These observations indicate that alternative materials are needed for microelectrode device fabrication and that the material properties of these devices are extremely important for long-term reliability and eventual integration into human therapeutic utilization through BMI or BCI applications.

While silicon continues to be one of the most prominent neural interface systems, we believe that its shortcomings can be greatly improved by replacing Si with silicon carbide (SiC). SiC is a material that is nearly chemically inert, only showing dissolution to potassium hydroxide at 400 °C [22]. It is a robust material with increased resistance to fracture compared to silicon [23,24]. Most importantly, it has multiple material forms, from the insulating amorphous form to several conductive single-crystalline forms [25,26]. Silicon carbide has experimentally demonstrated excellent cellular and tissue compatibility [27,28], hemocompatibility [29,30], electrochemical potential [31], and benefits from many processing techniques used in silicon chip fabrication [32]. This review will look at many of the devices that have used SiC for implantable neural electrode devices to provide the reader with a comprehensive review of this promising neural interface technology.

## 2. SiC Neural Interfaces

### 2.1. Amorphous Silicon Carbide (a-SiC)

Amorphous silicon carbide (*a*-SiC) has already been successfully adopted for use in many human biomedical devices, ranging from dental implants, bone replacement coatings, and heart stent coatings [33,34,35]. This ceramic-like coating retains all the characteristics of silicon carbide that make it an attractive material for biomedical devices. It possesses excellent chemical resistivity, has demonstrated excellent mechanical integrity, and has shown excellent biocompatibility [36,37]. Additionally, this material is electrically insulating, with a variable dielectric constant ranging from ~2.7 to 14, which is achieved by changing the stoichiometry, pressure, and temperature during deposition [38,39]. Another major advantage of *a*-SiC is that it can be deposited at much lower temperatures than single-crystalline SiC through the assistance of plasma-enhanced chemical vapor deposition (PECVD), allowing it to be used with high-temperature polymers like polyimide [40]. It follows that *a*-SiC may be a material that could improve the reliability of implantable neural interfaces.

#### 2.1.1. Utah Intracortical Array (UEA)

Solzbacher et al. reported coating the Utah Intracortical Array (UEA) with *a*-SiC to improve its tissue compatibility [41]. In this work, hydrogenated amorphous silicon carbide (*a*-SiC*_x_*:H) films deposited by PECVD using SiH_4_, CH_4_, and H_2_ precursors were investigated as a protective encapsulation layer over an integrated UEA. The encapsulation required that the *a*-SiC have a deposition temperature of less than 200 °C to protect polymeric components, and it needed to provide a uniform conformal coating while maintaining high electrical impedance with a low dielectric constant. While this is a very positive step towards a long-term human interface, it should be noted that the heterogeneously integrated *a*-SiC material added many issues. The PECVD-deposited *a*-SiC demonstrated a large amount of compressive stress, a known issue for deposition temperatures less than 400 °C, but the stress is usually relieved through thermal annealing, which unfortunately occurs at high temperatures exceeding 500 °C [42]. However, by modifying silicon to carbon ratios and hydrogen dilution through experimentation to a precursor ratio of 0.17 and hydrogen dilution of 5.71, they found a low-stress *a*-SiC, which retained a high level of Si-C bonding. These films had a dielectric constant of 4.5 and could be deposited at 200 °C. While these films passed a soaking test without measurable dissolution or development of defects like pinholes, the coating was far from uniform, tending to be thicker along the tips of the spikes and decreasing rapidly to their base.

#### 2.1.2. *a*-SiC Planar Chronic Cortical Array

Recent works have been reported by the groups of Joe Pancrazio and Stuart Cogan [37,43,44,45], which further demonstrate the benefits of using *a*-SiC for chronic neural interfaces. Unlike the study by Solzbacher, these chronic neural implants did not use *a*-SiC as a protective coating, but as the major component of insulation and to provide mechanical integrity. First, 1 μm of polyimide was deposited as a release layer, followed by an *a*-SiC base layer. The Ti/Au/Ti traces were then encapsulated with a top layer of *a*-SiC, and sputtered iridium oxide (SIROF) was used as the electrode. The final devices have a very thin, minimally invasive cross-section, as compared to commercially available planar electrodes, with a width of 20 μm and thicknesses starting at 6 μm in their initial study [46], increasing to 8 μm in subsequent studies [43,44,45] to alleviate buckling issues while maintaining low levels of flexural rigidity.

#### 2.1.3. Experimental

**Figure 1:**
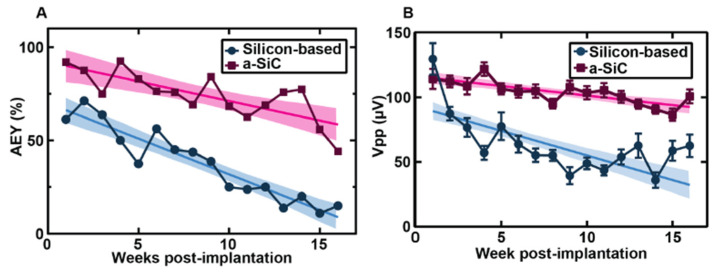
Comparison of silicon-based and *a*-SiC MEA performance. (**A**) Comparison between the active electrode yield (AEY) of silicon-based (blue circles) and *a*-SiC (pink squares) MEAs weekly over the 16-week chronic study with 95% confidence intervals and regression line. At week one, the AEY are significantly different (*p* < 0.0001). (**B**) Comparison between silicon-based and *a*-SiC MEAs’ peak to peak voltages (Vpp) with SEM, 95% confidence intervals, and regression line [45].

**Figure 2:**
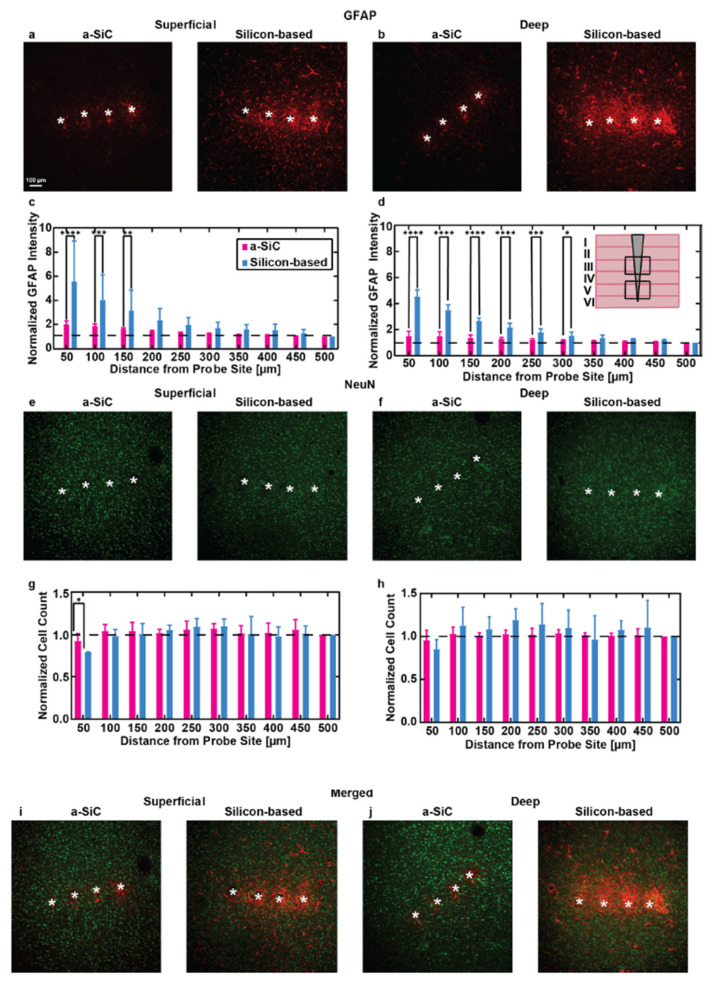
(**a**,**b**) Representative images of brain sections stained for glial fibrillary acidic protein (GFAP), showing immune responses at superficial (100–800 µm) and deep (800–1200 µm) depths below the pial surface for *a*-SiC and silicon-based probes. (**c**,**d**) Normalized GFAP intensity (mean ± SEM) as a function of distance from the probe site, comparing the immune response to *a*-SiC and silicon-based probes (mean ± SEM). The inset diagram in (**d**) demonstrates the implant location relative to the cortical layer. (**e**,**f**) Brain sections stained for neuronal nuclei (NeuN) in superficial and deep layers near both probe types. (**g**,**h**) Normalized NeuN cell count at increasing distances from the probe sites, comparing neuron density between *a*-SiC probes (pink) and silicon-based probes (Blue). (**i**,**j**) Merged images of GFAP and NeuN staining in superficial and deep brain layers for both a-SiC and silicon-based probes. White asterisks represent the identified implant holes. The statistical significance is represented by black asterisks (* *p* < 0.05, ** *p* < 0.01, *** *p* < 0.001, **** *p* < 0.0001) [45].

In three separate studies, the *a*-SiC planar arrays were implanted into the motor cortex of female Sprague–Dawley rats, and weekly, electrical and electrochemical recordings were performed for 16 weeks after implantation. Additionally, two of the studies compared the *a*-SiC electrodes against similar commercial silicon planar probes (A4 × 4-2 mm-200-200-200-CM16LP, NeuroNexus Technologies, Ann Arbor, MI, USA) [43,45]. In comparison to the recording of neural signals, the expression of single units measured from these *a*-SiC devices was significantly higher than for silicon-based MEAs throughout the 16-week implantation period (Figure 1), with the *a*-SiC electrodes remaining near 50% available electrode yield (AEY) at week 16 [44,45]. When recording local field potentials (LFP), there was a significant loss in spectral bandwidth power to nearly 20 dB below the initial conditions for silicon, while the *a*-SiC devices only lost 3 dB at the end of the study [43]. Electrochemical impedance for the *a*-SiC SIROF (sputtered iridium oxide) electrodes increased slightly over the time of implantation, but the change in magnitude was considered insignificant to the neural signal recordings and was comparable to studies made for SIROF electrodes using polyimide and silicon [44]. Additionally, the cathodal charge storage capacity did not show a significant change over the 16-week period, indicating that the *a*-SiC insulation did not experience significant degradation in vivo [44]. An immunohistochemical analysis demonstrated a greatly reduced neuroinflammatory response due to the a-SiC MEAs’ presence, as compared to the commercial silicon-based devices [Figure 2] [45]. The *a*-SiC implants also demonstrated a higher retention of neurons around the recording sites. While they attributed contributions from the lowered immune response to the differences in the relative size of the device shanks (8 μm thick and 20 μm wide for the *a*-SiC devices, as compared to the 15 μm thick and 45 μm wide silicon planar devices) and their relative flexibility (6.4 × 10^−11^ N·m^2^ for the *a*-SiC shanks and 240 × 10^−11^ N·m^2^ for the silicon shanks), they do show that *a*-SiC is a promising candidate for chronic neural implants. In addition, the *a*-SiC material’s physical strength allowed for the construction of small cross-sectional area neural implants that avoid having to use large rigid shuttles to be able to implant within the neural cortex.

### 2.2. Carbon Electrodes on a-SiC

Barrese et al. demonstrated issues with metal electrodes chronically implanted for up to 1051 days [21], which included corrosion and delamination. While platinum and iridium are considered insoluble and stable in physiological electrochemical environments [47], the oxidative species released during the brain’s inflammatory response are believed to lead to the corrosion of metals [18,21,48]. Carbon, a highly versatile element in the periodic table, has been widely integrated into various device technologies due to its electrically conductive properties [49]. Since 2000, carbon-derived materials—including carbon nanotubes [50], graphene [51], glassy carbon [52], and pyrolyzed photoresist film (PPF) [53]—have gained prominence in biomedical applications, particularly showcasing promise in microelectrode-based implantable neural probes (mINP) [54,55]. Correspondingly, PPF showcases superior biocompatibility [54,56,57], electrical conductance, structural resilience, and chemical passivity [54,58]. Materials that display a reduction in biotic reactions could increase the reliability of chronic neural implants, and carbon could become another material with which to achieve this goal.

Feng et al. developed a planar neural implant using high-conductivity carbon (C) conductors and *a*-SiC insulation material [59]. The C layer was fabricated by patterning photoresist using current microelectronic fabrication techniques, which were then followed by a baking step within a high-temperature furnace. Additionally, using this type of C layer fabrication not only allowed us to replace the metal electrode, but also the metal conductive traces. Details on the development, fabrication, and characterization of the carbon/*a*-SiC hybrid neural interface are included in the next section.

#### 2.2.1. Fabrication

**Figure 3:**
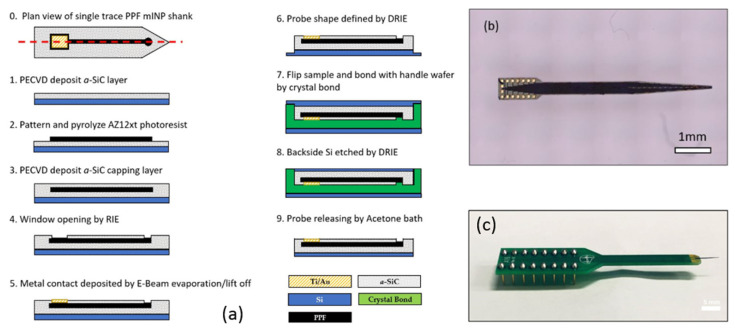
Flexible C/*a*-SiC electrodes: (**a**) Schematic of *a*-SiC/PPF hybrid probe fabrication process. (**b**) Optical microscope image of released *a*-SiC/PPF hybrid neural probe. (**c**) Final packaged device bonded to a PCB header for electrical testing [59].

The C neural probe process started with the deposition of a 250 nm thick film of *a*-SiC on a Si wafer via PECVD (360 sccm CH_4_, 12 sccm SiH_4_, 700 sccm Ar, 250 °C, 1100 mTorr, 200 W). The wafer was then RCA-cleaned and functionalized with HMDS. AZ-12XT-20PL photoresist (MicroChemicals GmbH, Ulm, Germany) was spun to form a 7 μm thick photoresist film, and photoresist traces were patterned through ultraviolet exposure. Pyrolyzation was then performed by annealing the photoresist in a tube furnace (900 °C, Ar 100 sccm, H_2_ 5 sccm) for 60 min. The traces were then capped with another layer of insulating *a*-SiC. The upper layer of *a*-SiC was selectively removed to produce both recording site windows and bond pad connections via reactive ion etching (RIE) (CF_4_ 37 sccm, O_2_ 13 sccm, 200 W, 50 mTorr). Metal lift-off filled in the bond pad areas with 50 nm of Ti, followed by 300 nm of Au deposited in an electron beam evaporator. The devices were then defined using deep reactive ion etching (DRIE), and the entire wafer was thinned to a 50 μm thickness. Figure 3a details the complete process described above and shows a single probe (Figure 3b) that was packaged with a printed circuit board and a DIP pin set (Figure 3c) for electrochemical evaluation.

#### 2.2.2. Experimentation

Electrochemical evaluation in phosphate-buffered saline (PBS, pH 7.4) was performed using a three-electrode configuration comprising the working electrode (pyrolyzed photoresist film, PPF), a counter electrode (platinum, Pt), and a reference electrode (silver/silver chloride, Ag/AgCl). Both cyclic voltammetry (CV) and electrochemical impedance spectroscopy (EIS) measurements were performed. EIS measurements were performed with a frequency sweep from 10 Hz to 100 kHz, with data acquisition at 10 points per decade. CV experiments were conducted within an empirically defined safe potential window of −0.6 V to 0.8 V, employing a scan rate of 50 mV/s, and repeated for three consecutive cycles. The complex electrical impedance of the electrodes was determined via the EIS measurement, while the charge storage capacity (CSC) was determined by integrating the CV data.

**Figure 4:**
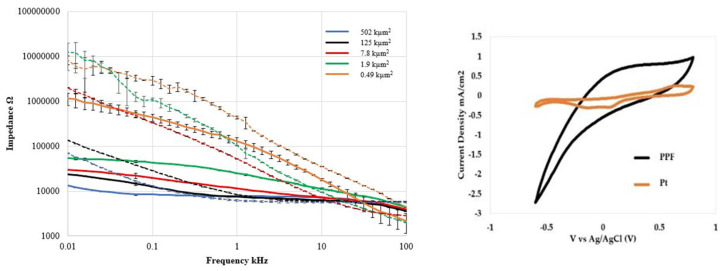
Electrochemical characterization of PPF (C) and Pt electrodes vs. recording tip surface areas: (**Left**) Electrical impedance spectroscopy vs. frequency and (**Right**) Cyclic-Voltammetry (CV) comparison of PPF and Pt (surface area = 502 kµm^2^). Note the significantly larger curve area for PPF electrode indicating superior charge storage capacity (CSC) [59].

While the study did not provide any biological interaction, the *a*-SiC/C hybrid probe demonstrated excellent electrochemical charge storage capacity (14.16 C/cm^2^), low electrical impedance (24.8 ± 0.4 kΩ @ 1 kHz w/surface area = 1.9 kµm^2^) (Figure 4). Table 1 compares the 1 kHz impedance and charge storage capacity of commonly used electrode materials with the measured pyrolyzed C electrode performance from this study. The study not only proves that pyrolyzed photoresist is a promising electrode material, but it could also be used to replace the metals used for the trace wires and further opens possible candidate materials for a reliable chronic neural implant.

### 2.3. Monolithic Hexagonal Silicon Carbide (4H-SiC)

The previous sections have presented evidence that amorphous silicon carbide (*a*-SiC), with its robust physical nature, chemical inertness, and excellent, customizable dielectric properties, may contribute to increasing the chronic reliability of an implantable neural device. Neural implants that contain *a*-SiC materials have demonstrated a lower biotic response, lasting up to at least 16 weeks in vivo [43,44,45]. However, many of the issues that have plagued the longevity of many chronically implanted neural interfaces arise from abiotic issues [17,18,19]. The neural implant devices presented in the previous sections have relied on multiple, heterogeneously integrated materials to provide the conductive, insulating, protective, and mechanical properties necessary for a functional neural implant device. Water uptake by polymers has led to physical swelling and increased stress between adjacent heterogeneous layers and has contributed to the exposure of protected materials due to cracking. Delamination due to inadequate surface adhesion has also been one of the leading device failure mechanisms [19]. Unfortunately, *a*-SiC has also experienced known issues due to inadequate surface adhesion, especially concerning the interface with metals, which are the prime conductive component for contemporary neural interfaces [38,39,40]. While titanium can be used as an adhesion promoter between *a*-SiC and noble metals like gold, it requires high-temperature annealing to facilitate adhesion, which can not only introduce stress in the *a*-SiC layer but can also damage low-temperature materials, especially polymers. Ti also alloys with other metals, like Au, leading to a sharp decrease in conductivity. One way to reduce the abiotic issues would be the utilization of a single, homogeneously integrated material that would provide excellent chemical resilience, physical robustness, and electrical properties. Using such a single material would reduce delamination and stress due to heterogeneous material junctions, leading to longer device lifetime in vivo.

SiC in its crystalline form is a semiconducting material, and, like the more familiar semiconductor silicon (Si), it can be doped with specific atoms to increase the material’s electrical conductivity. Dopants can either donate excess electrons, thus forming n-type (negative net charge) regions, or accept electrons, causing an electron deficit in the crystal, thus forming p-type (positive net charged) regions. Crystalline SiC also has another interesting property that makes it an uncommon semiconductor: polymorphism [60]. SiC has many crystalline forms arising from various atomic lattice geometries, such as cubic, hexagonal, or rhombohedral. When combined with atomic lattice stacking, this results in the formation of crystals with different electrical properties. Today, the most prevalent polytype is 4H-SiC, a hexagonal form of SiC with four repeating stacking sequence layers. 4H-SiC has one of the widest band gaps of the SiC crystalline family, 3.3 eV, which is the width of the energy range where no electronic states exist and can be purchased commercially in bulk wafers. Perhaps the most important point is that numerous in vitro and in vivo tests of various SiC crystalline forms have consistently demonstrated excellent biotic performance [61], thus suggesting that this material system is possibly a good choice for realizing long-term implantable neural interfaces for human application.

4H-SiC, as a semiconductor material, enables the fabrication of a homogenous neural implant device, or a device that uses only a single material for its fabrication. To understand how this would be possible, one must understand the concepts behind the pn junction [62]. The pn junction is formed when a positively doped material (P) is in contact with a negatively doped material (N). This creates a depletion region, which creates a barrier to electronic conductivity. The depletion region is created when the majority carriers from the doped materials diffuse into the oppositely charged material, which generates a diffusion current comprising both electrons and holes. The depletion region reaches thermal equilibrium when a built-in electric field, formed by the ionized dopant atoms in the depletion region, creates a drift current that counteracts the diffusion process. One important property of semiconductors occurs when a region is heavily doped. This results in semi-metallic charge transport, meaning that sufficient conductivity is present, such that the material mimics metal conductors. Thus, one no longer requires metals, with all their known deleterious interactions in vivo, to achieve electrical signal routing on a neural device. When one appreciates that SiC is essentially a ceramic, meaning it has excellent physical and chemical stability, it then becomes clear that replacing metals with heavily doped (n^++^) traces is an option for neural signaling within the device.

One very important property of the pn-junction is that it acts as a diode that blocks current flow in one direction. This allows for the formation of adjacent conductive n^++^ traces on a p-doped substrate, which, when n^++^ mesas are formed, prevents ‘cross talk’ between parallel traces, thus obviating the need for an electrical insulator between the traces and substrate, which is needed in other Si-based neural interfaces. Therefore, by using the pn junction, the neural interface metallic conductor and underlying insulator can be replaced by doping the semiconductor material and forming long pn junction mesa diodes. However, one hurdle for this concept limits its efficacy, especially if one wanted to use the neural implant for stimulation. Applying an electrical bias to the P-doped side of a pn junction lowers the junction’s electrical barrier by neutralizing opposite charges, and at a sufficient level, allows for conductivity between the doped layers. However, applying a bias to the N-doped material would further increase the size of the junction by pushing away more of the conductive carriers and increasing the minority electrical field. Therefore, it would be imperative that the P-doped semiconductor material be used to replace the insulator material, and the N-doped material would then replace the conductive material to ensure that adjacent traces are electrically isolated during stimulation. This is easily achieved by forming electrical contact to the neural device via a contact pad to the n^++^ layer of the mesa diode.

Using homogenous semiconductor materials to replace the heterogeneous conductor/insulator material combinations in a neural implant device is possible due to the pn junction, but there is one final electrical issue that must be examined: the forward voltage drop [63]. This is a material property that is essentially the minimum amount of externally applied energy required to enable conduction in a forward-biased pn junction. For semiconductors like silicon and germanium, this forward voltage drop is around 0.7 V and 0.3 V, respectively [63]. The forward voltage drop may not be an issue with a neural implant that was used for recording only, as the bias levels would be in the μV range for action potentials and mV range for local field potentials. However, the forward voltage drop would limit the stimulation efficiency of neural implant devices constructed from semiconducting materials, especially when stimulated in the negative regime. If a heterogeneous Si device had −0.7 V applied during stimulation, the P-type material would be positively biased, as compared to the N-type material, allowing for electrical conduction to flow from the traces into the entirety of the device’s body. 4H-SiC has a much higher forward-voltage drop of around 2.2 V to 2.3 V, thereby allowing for a much higher potential limit for safe stimulation without risking electrical leakage to the device’s body. It is important to note that most stimulation voltage amplitudes are in the 1–4 V range, with most treatments starting with a 1 V amplitude. Due to the challenges addressed early, as the neural implant reacts with the local tissue, the voltage amplitude is increased to overcome the tissue reaction that surrounds the device. By using a tissue-compatible material like SiC, in combination with its ~2.2 V blocking voltage, one can envisage stable, long-term stimulation potentiality without the need to constantly adjust the stimulating voltage. This will be elaborated further in Section 3 of this paper. In this section, we report on a study by Bernardin et al., which used the pn junction concept to investigate the possibility of a homogeneous, single-material 4H-SiC neural device to replace the heterogeneous insulator and conductor materials in Si-based devices [31].

#### 2.3.1. 4H-SiC MEA and IDE Device Fabrication

**Figure 5:**
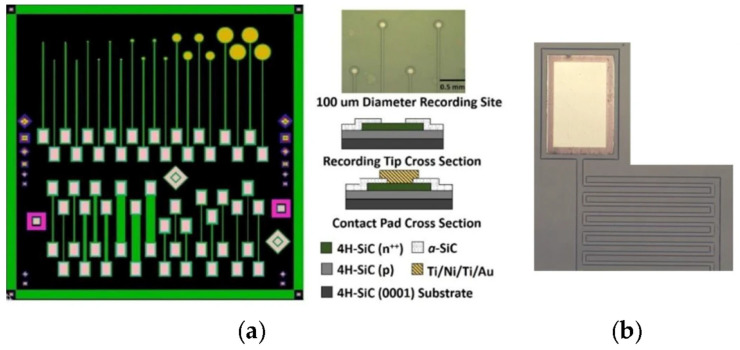
The 4H-SiC MEA device consists of single-ended electrodes with various recording areas, pn junction diodes, and resistors of assorted lengths and widths. (**a**) Mask layout for the MEA showing single-ended electrodes (top) and double-ended electrodes for resistivity, along with Schottky and pn diodes (diamonds and squares). To the upper right of the mask layout is an optical micrograph of the 100 μm diameter recording sites, and the lower right shows a cross-sectional schematic of the electrode and testing pads. (**b**) An optical micrograph of one of the 4H-SiC IDE devices showing a gold contact and pn junction mesa traces, which were isolated with a-SiC [31].

Figure 5 shows two devices designed by Bernardin et al. to investigate the homogeneous 4H-SiC electrode performance [31]. First is a planar microelectrode array (MEA) consisting of twenty single-ended planar electrodes with tip diameters of 25, 50, 100, 400, and 800 μm. These were chosen to evaluate and characterize the electrochemical effects relative to geometric surface area (GSA) of the electrode and the effect of the length of traces on the overall impedance of the device. The second device, an interdigitated electrode (IDE), was created to investigate the electrical efficiency of both the pn junction isolation and the conformal *a*-SiC insulator that would encapsulate an implantable device. These devices were created by using samples cut from a 4H-SiC epitaxial wafer that was grown to include a p-type base layer capped with an n^++^ semi-metallic layer. The samples were grown in a hot-wall CVD reactor, which was used to realize two epitaxial layers: first, a 5 µm p-type base layer was grown, followed by a 2.5 µm n^+^-type electrode layer, thus creating an integral pn junction. Deep reactive ion etching (DRIE) was used to dry etch ~3 µm into the epiwafer, thus removing the n^++^ layer and exposing the p-type base layer. Photolithography was used prior to DRIE to define the traces, bond pad areas, and electrodes, whereby thick photoresist served as the etch mask. Electrochemical isolation for the p-type and n-type electrodes was achieved through the addition of a 200 nm thick layer of *a*-SiC. The PECVD process conditions were 13.56 MHz, 250 °C, 900 mTorr, SiH_4_ 360 sccm, CH_4_ 12 sccm, and Ar 500 sccm. Photolithography was used to define the insulator windows, which exposed both the n^++^ 4H-SiC electrodes (i.e., recording tips) and the 4H-SiC mesa base tab for the formation of metal bond pads to facilitate wire bonding/packaging. Reactive ion etching (RIE) was used to remove the *a*-SiC film (CF_4_ 37 sccm, O_2_ 13 sccm, 200 W, 50 mTorr) selectively in these two places. The metal bond pads were then defined through a lift-off lithography process and included a thin, base layer of titanium (Ti), followed by a layer of nickel (Ni). These metals were annealed in a rapid thermal process (RTP) at 1000 °C for 30 s to facilitate an ohmic contact. A secondary lift-off process was performed using Ti and gold (Au) deposited directly onto the annealed Ti/Ni stack to create a surface suitable for wire bonding or electrical testing using probes. The final devices were then annealed at 450 °C for 30 min to alloy the bond pad metals, thus lowering their contact resistance. The exact same fabrication procedure was used to realize IDE devices with a 50 μm mesa width and a pitch of 25 µm.

#### 2.3.2. Experimental Results

**Figure 6:**
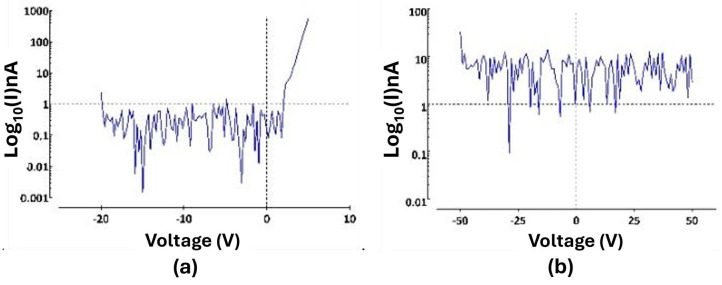
(**a**) A pn junction IV measurement of the IDE, showcasing a forward-voltage drop of the pn diode of ~2.3 V and a leakage current below 1 nA through −20 V. (**b**) IV measurement (−50 to 50 V) amongst neighboring traces showcasing minimal crosstalk between two IDE traces [31]. Note that this is effectively a measure of the leakage current from an N-P-N junction and clearly shows current blocking from −50 to +50 V.

Twenty interdigitated electrode (IDE) devices were used to evaluate the pn junction and *a*-SiC insulation properties of the 4H-SiC electrode devices. Current–voltage (IV) measurements were utilized to establish the forward-voltage drop for the pn junctions (−20 V to 5 V increasing at 0.5 V/s) and to determine the pn junction isolation between adjacent electrode traces (−50 to 50 V increasing at 5 V/s). Figure 6 displays data obtained from one of the 4H-SiC IDE devices, which was representative of 14 of the 20 IDE devices tested [31]. As was expected, due to the previous studies of 4H-SiC pn diodes, the forward voltage drop for the devices was close to 2.3 V. The isolation between electrodes was slightly more varied, with no appreciable leakage current (<1 nA_rms_) for fourteen of the IDE devices from −50 to +50 V. Of the remaining six devices tested, four devices still blocked voltages near the tens of volts, while two demonstrated Ohmic flow at voltage potentials of −2.3 V. In all cases, no appreciable leakage current was observed in the −2.3 to +5 V range, indicating sufficient isolation for stimulation within this voltage range.

**Figure 7:**
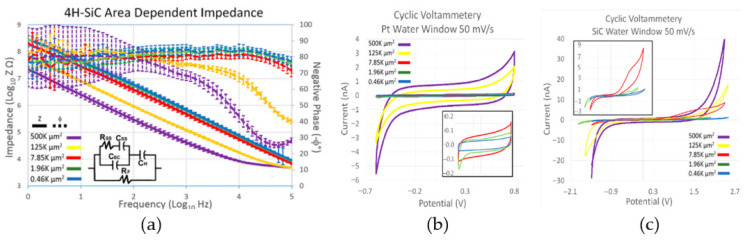
(**a**) EIS data plot of 4H-SiC electrodes with different geometrical surface areas. (**b**) CV plot of the single electrodes (**a**) swept through a platinum water window (−0.6 V and 0.8 V) (**c**) and swept through the SiC water window (−2.0 V to 2.7 V) [31].

Electrochemical tests, namely EIS and CV measurements, were performed in phosphate-buffered solution (PBS, 7.4 pH, 22 °C). A three-electrode setup was employed, featuring a 127 μm diameter, 5 cm long Pt wire, a 254 μm diameter, 5 cm long Ag/AgCl reference electrode, and a 4H-SiC working electrode. EIS and CV tests were conducted on four different samples, each with 20 electrodes in five distinct sizes. EIS involved applying a 10 mV sinusoidal wave across a frequency range of 0.1 Hz to 100 kHz, with the current response recorded 12 times per decade. Initial CV measurements were swept through potential limits of −0.6 to 0.8 V (50 mV/s), corresponding to the well-known platinum water window. A second set of CV measurements was performed, but the potential limit was widened until the current limit was reached to evaluate the width of the 4H-SiC water window. The electrochemical interaction of the 4H-SiC electrodes with PBS demonstrated purely capacitive characteristics, as demonstrated with the phase angles of ~80 °C (Figure 7a). At 1 kHz, the frequency associated with cortical action potentials, the electrodes with an area of 496 μm^2^ had an average impedance of 675 ± 130 kΩ, which would work with commercially available neural recording electronics (Figure 7a). The electrodes demonstrated reasonable linearity with a decrease in impedance associated with an increase in geometrical surface area (Figure 7a).

The cyclical voltammetry (CV) measurements using the platinum water window (Figure 7b) further reinforced that 4H-SiC operates electrochemically using capacitive charge transfer. The current charges to a saturation level and remains relatively constant until the voltage is reversed. An increase in geometric area increased the current limit for the electrodes. The study’s electrochemical modeling postulated that the electrodes were mainly dependent on charge transference through capacitive conductivity, a capacitance which was primarily dominated by a depletion layer that develops inside the surface of the highly doped n-type semiconductor at the electrolyte boundary. This is a zone that has a depletion of the majority charge carriers, as was the case with the pn junction, but is instead caused by the electrolytic ions attracted or attached to the semiconductor surface. This layer had a smaller capacitance when compared to the Helmholtz capacitive layer, a capacitive layer developed by the water attracted to the semiconductor surface, and as capacitors in series with each other, the overall capacitance was dependent on the much smaller surface depletion junction capacitance. The evaluation of the 4H-SiC water window demonstrated a water window from −2.0 to 2.8 V (Figure 7c), which is significantly larger than most of the common materials used for neural implant stimulation [31].

The conclusion of the electrochemical study indicates that 4H-SiC may be an excellent single, homogenous material that could be used to fabricate a reliable cortical neural implant. The impedances are on par with traditional metallic electrodes used in cortical neural implants. However, the material uses capacitive transfer instead of Faradic transfer, which is used by metallic electrode materials [41]. This capacitive transfer could add issues when used as a stimulation electrode. The most relevant would be the time required for capacitive charging and discharging, which would affect high-frequency stimulation. It was also noted that the charge storage of 4H-SiC was comparable to materials like Pt; it was very low when compared to PEDOT, carbon, and iridium oxide. The latter materials use Faradic transfer and have generally been considered the gold standard for electrochemical stimulation. One improvement that was postulated by Bernardin et al. was increasing the doping density of the n-type 4H-SiC, as highly doped diamonds had shown a large charge storage capacity under these conditions [64]. One advantage that 4H-SiC also had was that the wide water window allowed for more charge delivery through the increase in potential and current. Overall, the study demonstrated that 4H-SiC could be used to fabricate isolated traces with low leakage and electrodes that could both stimulate and record from neural tissue. By using this single-material technique, the *a*-SiC layer could also be replaced by another epitaxial p-type layer to create a single-crystal, monolithic 4H-SiC device.

### 2.4. Cubic Silicon Carbide (3C-SiC) on Silicon on Insulator (SOI) Neural Implants

The previous section detailed the fabrication and electrical characteristics of a monolithic, homogenous neural implant constructed from 4H-SiC. 4H-SiC, a hexagonal form of SiC, was chosen for its wide bandgap and the commercial availability of large-area substrates. This study only demonstrated the electrical and electrochemical characterization of the devices and never presented the actual activity of 4H-SiC in vivo. Beygi et al., from the Saddow group, reported in their study that 4H-SiC substrates are expensive and can have many limitations for fabrication processes, mainly attributed to the material’s chemical and mechanical resilience [65]. Ironically, these are the very properties that make the material desirable for long-term operation within the harsh biochemical environment of the human body.

One of the biotic issues that has been attributed to reducing the reliability of neural implants stems from the physical and geometric size of the implant. The size is an important factor relating to the degree of acute damage caused during implantation of the device, but is also a consideration for chronic inflammatory activity when linked with mechanical mismatch [66,67,68]. One thought that has been gaining precedence in reducing the device’s inflammatory response is ensuring that neural device width and thickness remain less than 10 µm [69]. One issue with these smaller dimensions is that devices experience critical buckling due to their long length, thereby requiring the assistance of external shuttles for implantation. These shuttles are much larger in size and cause large amounts of acute damage [12]. As seen in Section 2.1, the physical strength of SiC enables it to be fabricated into devices that have less than 10 µm thickness. The 4H-SiC MEA devices presented in Section 2.3 had an approximate thickness of over 300 µm, which would require excess substrate material removal to achieve the desired neural interface geometries. Two commonly used thinning methods are to use physical grinding or dry etching; however, without an etch-stopping layer, the final safe thickness is determined by a combination of internal stress, which determines the bow in the substrate, and sample-mounting efficacy. One method to etch to specific depths is by adding an etch stop. An etch stop is a layer of material that slows the etching rate such that over-etching is more easily avoided. SiC is nearly chemically inert, so, unlike silicon, which has multiple atomic elements that can be added via diffusion, in SiC, these dopants are either added directly through epitaxial growth or through ion implantation and activation. Using layers of p-type or n-type dopants results in negligible differences when used in SiC dry etching, but doping does show different etch rates using electrochemical etching [70]. Alternatively, an ion implantation process called smart cutting can be used to create an appropriately thinned 4H-SiC wafer substrate [71]. The thin SiC wafer is bound to a stack that includes both a layer of silicon dioxide and a silicon substrate wafer (SiC-SOI). Unfortunately, the SiC-SOI process is very expensive, time-consuming, and has limited availability [71].

Cubic silicon carbide (3C-SiC) is the only cubic polytype of silicon carbide, but this gives it one advantage: it can be grown epitaxially on other cubic crystals, such as silicon [72]. Silicon wafers are almost 100 times cheaper than 4H-SiC wafers, and 3C-SiC can be grown directly on both silicon and/or silicon on insulator (SOI) wafers instead of using the expensive process of creating SiC-SOI. Using commercially available Si and SOI wafers opens many avenues to the fabrication of a free-standing silicon carbide neural implant device.

Apart from having a different growth substrate, 3C-SiC has many similarities and differences when compared to 4H-SiC; 3C-SiC has a lower bandgap than 4H-SiC at 2.3 eV, which will affect the electrical behavior of the pn junction, but this value (1.12 eV) is still much larger than silicon, the other major semiconductor used in neural interface devices. Some advantages are that it has the same dopants as 4H-SiC, aluminum (p-type) and nitrogen (n-type), and will use many of the same processing techniques required for 4H-SiC, like DRIE. Beygi et al. took the experiences and lessons from the 4H-SiC electrode development and applied these principles to fabricating a single-crystal 3C-SiC neural implant device, which will be described in the next section [65].

#### 2.4.1. Cubic Silicon Carbide (3C-SiC on SOI) Neural Interface Device

**Figure 8:**
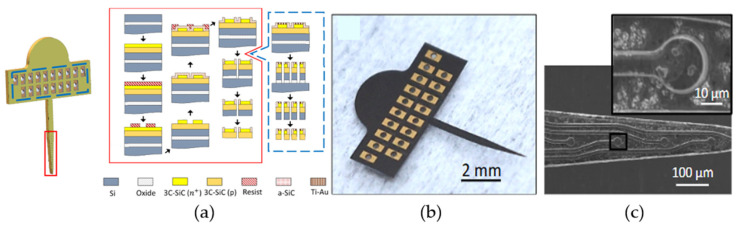
3C-SiC on SOI free-standing probes: (**a**) Processing flow for the probes, (**b**) photograph of the released 3C-SiC probe, and (**c**) SEM micrograph of the traces and recording sites (inset). The quality of the 3C-SiC was marginal, as evidenced by the surface defects observed under SEM [65].

Beygi et al. fabricated 3C-SiC neural probes using 3C-SiC epitaxial films grown on top of a conventional SOI substrate using low-pressure CVD (LPCVD). This wafer, a first attempt to grow high-quality 3C-SiC on SOI, was noted to have a high level of crystalline defects, with some regions being polycrystalline in nature. The purpose of this preliminary 3C-SiC on SOI development effort was to allow for ease of harvesting the final neural probes via wet-etching of a buried oxide layer (similar to what is performed for Si-based neural probes in the commercial marketplace), and it was understood that the electrical properties of the final probes would be affected by the poor crystalline quality of the 3C-SiC films. Nonetheless, processing of the neural probes was performed as follows:

The first layer was the 8 µm p-type base, and the second was a 2 µm n^+^-type conductive layer. Both layers had comparable doping levels compared to the 4H-SiC study. DRIE was used to etch through the n-type 3C-SiC down into the p-type layer, thus defining the n^+^ electrodes, traces, and bond pads. The entire wafer was coated with 250 nm of *a*-SiC grown via PECVD, which acted as the outer insulation for the devices. Windows were made into the *a*-SiC insulation to expose both the electrodes and the bond pad base layer. Next, metal (Ti/Au) was evaporated onto the bond pads and then annealed to create an Ohmic contact as well as strengthen the attachment between the metal and the 3C-SiC surface. At this point, the 3C-SiC neural implant probe process deviated from the 4H-SiC process. The probe’s physical outline was etched using DRIE through the *a*-SiC, 3C-SiC, and Si layers down to the buried oxide layer (from the SOI wafer). The 3C-SiC probes were then released from the carrier Si substrate via a wet etch of the buried oxide layer using 49% HF. The probes were bonded to a Si handle wafer, and the backside Si was removed using a Si DRIE process, which has a faster etch rate than SiC. Figure 8b shows the released neural implant device constructed from 3C-SiC on SOI, which bears similar characteristics when compared to other silicon-based planar implants. The devices had a large upper tab area, where Omnetics connectors were bonded directly to electrical connectors for electrical testing. One notable issue also evident from Figure 8b was that the released 3C-SiC devices possessed unresolved internal tensile stress, curving the devices slightly upward [65]. Fortunately, this curvature was not enough to prevent final processing or testing.

#### 2.4.2. Experimental Results

The pn junction was characterized electrically using current–voltage (IV) techniques by ramping voltage between −10 V and 10 V at a rate of 0.1 V/s and recording the current. Electrochemical measurements were performed using a three-terminal configuration, between the working (3C-SiC) electrode, a Pt counter electrode, and an Ag|AgCl reference within a 7.4 pH-balanced phosphate-buffered solution (PBS). Electrochemical impedance spectroscopy (EIS) measurements were performed from 0.1 Hz to 1 MHz at twelve points per decade. Cyclic voltammetry (CV) was measured from −0.6 V to 0.8 V at a rate of 50 mV/s, with three measured repetitions. Charge storage was calculated by using the area of the CV measurements.

**Figure 9:**
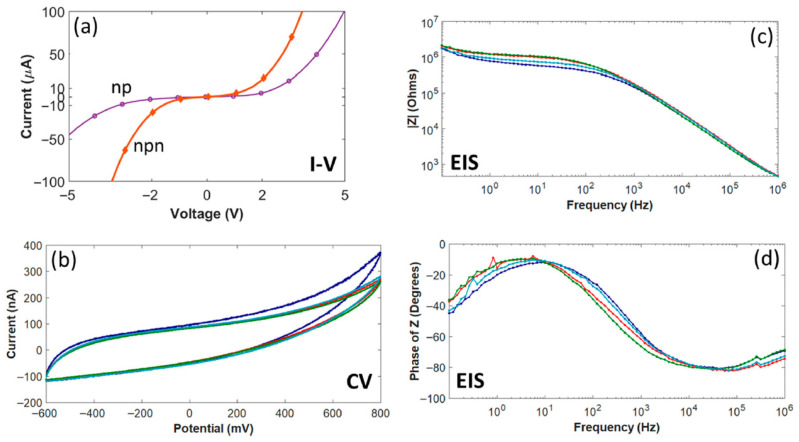
Electrical characteristics of selected 3C-SiC neural implant devices [65]. (**a**) IV data showing current flow from the N-type mesa to the P-type base (np) and between adjacent traces (npn), respectively. (**b**) EIS impedance amplitude data as a function of frequency. (**c**) CV data showing three (3) sweeps over a water window from −600 to + 800 mV. (**d**) EIS impedance phase data vs. frequency. Multiple colored traces represent repeated trials on the same device and demonstrate consistent electrode behavior.

As seen in Figure 9a, the forward voltage drop across the pn junction measured at 2 V, smaller than the previous study for 4H-SiC, but not unexpected due to the lower bandgap [65]. The true surprise was that the reverse breakdown voltage was much lower, at approximately −2 V. This value is not only lower than the previous devices fabricated in 4H-SiC, which were able to block up to −50 V, but was even lower than that seen in silicon pn junction diodes, which are typically below −5 V potential [63]. Furthermore, when the isolation between two adjacent traces was tested, a junction which is essentially an n-p-n junction, both the forward voltage drop and reverse breakdown potential lowered to 1.4 V and −1.4 V, respectively. One explanation given for this was attributed to crystalline defects that were generated during the epitaxial growth. 3C-SiC has a 20% lattice mismatch when compared with Si, and this mismatch leads to a misfit dislocation where an atom of the SiC crystal is not bonded one-to-one at the Si interface [73,74]. This defect propagates through the crystal, leading to a larger, linear defect, degrading electrical activity by eliminating electrons or holes through recombination. Finally, it was noted that the 4H-SiC leakage currents were on the order of nA, whereas the 3C-SiC leakage was in the µA range. The larger leakage currents would have a much larger effect on neural recordings, especially as they are in the order of tens of µV and nA.

3C-SiC demonstrated a capacitive electrochemical interface, much like its 4H-SiC counterpart. As shown in Figure 9b, the current peaks irrespective of the voltage and only changes during the reversal of potential directions [65]. Most interestingly, the charge storage capacity of a 491 µm^2^ 3C-SiC electrode was on the order of 15 mC/cm^2^, which was two orders of magnitude greater than the 4H-SiC electrode with 0.41 mC/cm^2^. The study only focused on the Pt water window and did not report the absolute water window for the 3C-SiC devices, as it did for the 4H-SiC. Impedance was similarly lowered, with 3C-SiC having a 1 kHz impedance of 16 kΩ when compared with 4H-SiC at 675 kΩ (Figure 9c) [65]. The decrease in impedance and increase in charge storage capacity were attributed in part to the greater surface area of the 3C-SiC surface. Unlike 4H-SiC, which has nm roughness, 3C-SiC possesses a rougher surface due to island nucleation growth during epitaxy and crystalline defects. While the 3C-SiC and 4H-SiC electrodes both have a similar diameter, the roughness of the surface increases the effective area that interacts with the electrochemical environment. Another reason could be due to the defects and leakage into the devices. Overall, the group demonstrated single-crystal 3C-SiC neural implant devices that were fabricated into physiologically relevant interactive sizes with observed electrochemical impedances on par with metal electrode neural interfaces.

### 2.5. Cubic Silicon Carbide MRI Compatibility

Neuromodulation has become an established therapy that assists with many neural disorders, such as Parkinson’s and Alzheimer’s, while Vagal stimulation has shown major promise as well [75,76]. Many of these diseases use magnetic resonance imaging (MRI), a noninvasive diagnostic technique that uses magnetic and electrical fields to image soft tissue [77]. However, neuromodulation devices are not necessarily compatible with the electromagnetic fields produced by MRI techniques [78]. The electrical operation of neuromodulation implants requires materials that conduct electrical current during both neural recording and stimulation, and many of these materials interact with both the magnetic and electric fields produced by the MRI. The electrical and magnetic fields induce electrical current within the devices, leading to artifacts that interfere with imaging, generate ohmic heating, and create erratic electrical stimulation to the tissue surrounding the implants. While the image artifacts interfere with diagnostic capability, the induced currents can cause detrimental stimulation or even cellular damage if the heating goes above 40 °C [79]. For individuals who have neural modulation implants, limiting the magnetic (B) field produced by the MRI to a level of 1.5 T to 3 T can alleviate many of these dangers, but this level of magnetic field is not optimal for high-resolution MRI, which often can be required for many neural disorders [80].

MRI using 7T or more is required to improve diagnostic techniques and create sufficient high-resolution for structural and susceptibility-weighted imaging [78]. There are many approaches to fabricating MRI-compatible devices, including using low-conductivity materials, materials with low magnetic susceptibility, shielding materials, and coiling techniques [81,82,83]. Magnetic susceptibility has been postulated as one of the most important factors in image artifacts, while electrical conductivity is important for the induction of electrical current [82,84].

Beygi et al. postulated that a homogenous 3C-SiC neural implant device would be advantageous for MRI compatibility when interacting at a higher resolution, such as at 7T [85]. 3C-SiC has a wide array of material properties that enable it to interact with high-level magnetic and electrical fields. It has a thermal conductivity on par with copper and a magnetic susceptibility compatible with brain tissue. 3C-SiC has a magnetic susceptibility of −12.87 ppm, which is very close to human tissue (−9 ppm), and is lower than that of platinum (267 ppm) and silicon (−3.24 ppm) [86]. Image artifacts are due to magnetic field perturbation occurring when a material has a different magnetic susceptibility than that of the tissue [84]. Additionally, while 3C-SiC has been doped to conduct electrical current, the lower electron concentration of highly doped 3C-SiC, along with lower electron mobility and lifetime, decreases the level of induced current, reducing Ohmic heating in the neural probe’s traces.

The 3C-SiC neural probe discussed above was placed into a saline-based brain gel phantom, and sagittal and coronal scans were made. This was performed with Si and metallic controls, and only the 3C-SiC probes evidenced no MRI artifacts. Unfortunately, low-resolution temperature sensors made it impossible to measure any temperature rise due to the low resolution of the fiber-optic temperature sensors available (±1 °C). While this may indicate that tissue heating of less than 1 °C was present, future experiments are needed to validate this claim. More importantly, in vivo animal studies are needed to truly assess the MRI compatibility of 3C-SiC neural probes, and these experiments are pending. Nonetheless, these experiments indicate that an all-SiC neural probe may indeed be MRI compatible and therefore suitable for use in conjunction with ultra-high field magnetic resonance imaging. The study by Beygi et al. [85] compared various materials along with fully fabricated 3C-SiC neural probes inserted into a saline gel phantom and exposed to the electromagnetic fields produced by a small animal 7T MRI. Computer simulations for the electromagnetic and thermal interactions of various materials in a 7T MRI were also made. The results of the simulation and data collected from the electromagnetic field interactions were compared to determine the activity of the tested material stacks within the high-power MRI field.

#### 2.5.1. Sample Fabrication and Simulation

**Figure 10:**
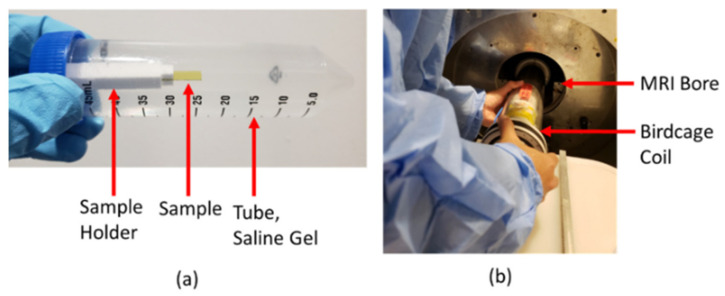
(**a**) 50 mL centrifuge tube containing an embedded sample w/holder in a gel phantom. (**b**) Photograph of a birdcage containing the sample from (**a**) being placed into the 7T MRI bore [85].

In addition to the free-standing neural probe from the previous section (Figure 8b [65]), eleven sets of simple rectangular slabs (15 mm × 4 mm) were tested within the 7T MRI tool [85]. 3C-SiC was epitaxially grown as described in the previous section, alternating between p/n^+^ and n^+^/p pn isolation, and traces were fabricated from the top layer. These samples were grown on SOI or n-type silicon. SOI was left on both 3C-SiC configurations for the first two samples, n^+^/p was left on n-type Si for the third, and the Si backing was removed for the next two tested samples. The remaining samples consisted of either plain substrates consisting of polyimide, n-type and p-type silicon, or these same substrates with platinum traces added. Each of the samples was mounted onto a 3D-printed polylactic acid (PLA) sample holder and placed within a 50 mL plastic tube (Figure 10a) [85]. The tube was filled with a mixture of polyacrylic acid (PAA) and sodium chloride (NaCl) that had a conductivity of 0.5 S/m at 300 MHz, a relative dielectric constant of 70, a diffusivity of 1.3 × 10^−7^ m^2^/s, and a heat capacity of 4150 J/Kg-C, which compares to the electrical and thermal properties of human brain tissue. These samples were mounted within a 35 mm M2M birdcage coil and then inserted into a 7T MRI (Agilent-Technologies w/Bruker electronics, Santa Clara, CA, USA) (Figure 10b) at the Moffitt Cancer Center on the USF campus. Coronal and sagittal T2-weighted images of the devices were captured using a RARE (rapid acquisition with relaxation enhancement) fast imaging sequence to assess image artifacts (TR/TE 2220/32 ms, 9 slices w/slice thickness 0.3 mm, FOV = 35 mm × 35 mm and 256 × 256 pixels).

Simulations for image artifacts and heating were also made for comparison with the acquired experimental data. Magnetic perturbation, which is a major factor in image artifacts, was simulated using MATLAB (R2017b, Math-Works, Natick, MA, USA). Samples of Pt, free-standing 3C-SiC, and Si substrates were used in conjunction with their magnetic susceptibility difference (Δχ). The samples were simulated in a saline-based gel phantom. The simulations generated the B_0_ field perturbation induced by each material. The specific absorption rate (SAR) under the RF excitation was calculated using ANSYS HFSS (19.2, Ansys, Inc., Canonsburg, PA, USA) through finite element method (FEM) simulation. The birdcage resonator at 300 MHz with the sample, holder, and tube was simulated to determine the B_1_ field distribution as well as the SAR. Due to the difficulty of measuring the actual thermal effects on the small samples, SAR evaluation, two fiberoptic fibers were mounted within the tube without the sample present to measure the temperature of the saline gel during 15 min of MRI exposure. The experimental results were then compared to the simulation to evaluate its accuracy.

#### 2.5.2. Experimental Results

**Figure 11:**
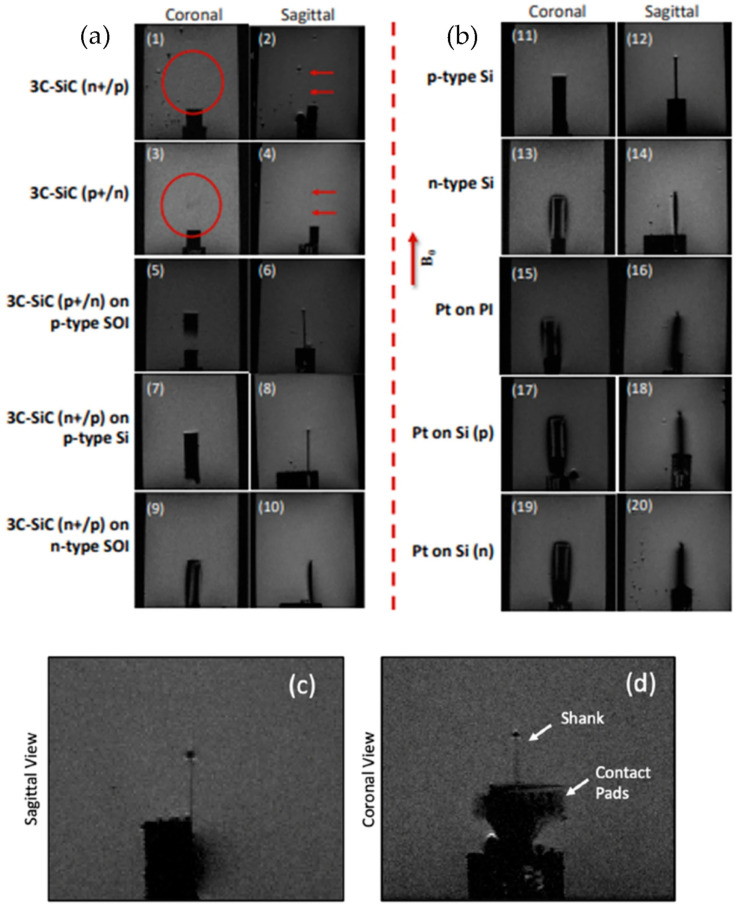
7T MRI images of different semiconductor materials: (**a**) (1, 2) 3C-SiC (n^+^/p) is almost invisible in the scan. (3, 4) 3C-SiC (p/n^+^) similar to (1, 2). (5–8) Minimal artifacts, yet visible samples, for 3C-SiC on p-type SOI and Si substrates. (9, 10). Artifacts and samples are visible for 3C-SiC on n-type SOI substrate. (**b**) (11–14) Artifact differences between n- and p-type Si substrates. (15–20) Severe image artifacts for all platinum samples. (**c**,**d**) The sagittal and coronal views of the 3C-SiC probe, showing a low level of artifacts except at the tip of the device and the area where the gold bond pads are located, which would not be within the tissue [85]. The red circles and arrows in (1) to (4) show the location of the probe which is barely visible in the images.

Figure 11 displays the RARE images obtained from the physical samples and 3C-SiC neural implant device when exposed to 7T MRI [85]. Both free-standing 3C-SiC slabs show almost no appreciable image artifacts within the field, but when these slabs are incorporated on top of SOI or n-type silicon substrates, they possess similar artifact generation, as seen with the bare Si slabs. Interestingly, the p-type and n-type Si slabs displayed very different artifact generation, with p-type silicon demonstrating a lower artifact profile than that produced by n-type silicon, suggesting that the doping type may influence the artifact intensity. Alternatively, this same intensity difference is not present in the free-standing 3C-SiC. Pt produces a large artifact irrespective of the substrate. Unlike the free-standing 3C-SiC substrates, the neural interface device shows a low level of image artifact generation along the shank and a larger level at the back end. The back of the device has Au metal pads to facilitate connection to an Omnetics connector, but this region would not be within the neural tissue; thus, this observation is not a concern. The shank, however, does have a darker outline. The authors attribute this artifact to the thin silicon film remaining under the 3C-SiC device. The overall thickness of the device was 27 μm, while the pn 3C-SiC stack is only 10 μm, with the conclusion that some silicon substrate has remained after processing the devices. The three Fourier-based simulations on 3C-SiC, Si, and Pt correlate closely to the data obtained from the actual MRI interactions, while the effects can only be generally compared, as the tested samples were multiple material stacks.

**Figure 12:**
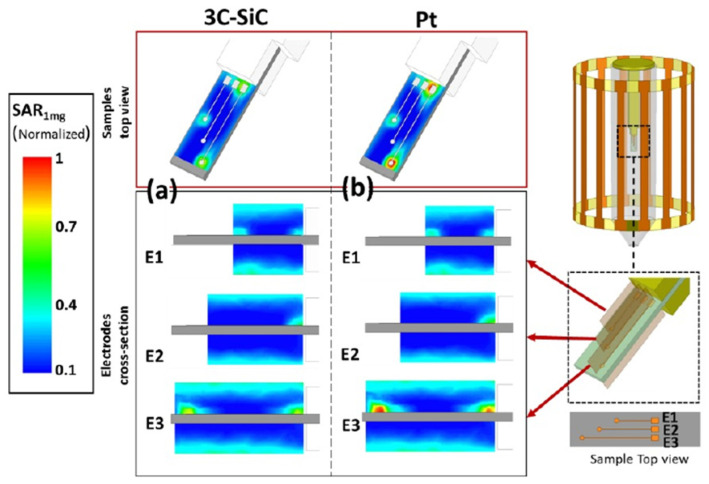
Simulated normalized SAR_1mg_ around the Pt and 3C-SiC (both on Si substrate) samples containing three electrodes. E1, E2, and E3 denote short, medium, and long electrodes, respectively. The results are normalized with the maximum SAR_1mg_ value that occurs in the vicinity of the largest Pt electrode. (**a**) Estimated normalized SAR_1mg_ for 3C-SiC via FEM electromagnetic simulations in ANSYS HFSS. Both top (top image) and cross-section (bottom images) views for each electrode are illustrated. Maximum estimated SAR_1mg_ for 3C-SiC appeared at the tip of the longest electrode, while the average B_1_ at the coil isocenter was ~10 mT. (**b**) Estimated normalized SAR_1mg_ distribution for the Pt sample [85].

Temperature measurements of the phantom gel were used as validation of the device simulation relating to heat generation and SAR. The phantom gel increased in temperature to 29.1 °C, as recorded by one of the fibers, and to 28.5 °C at the second sensor after 15 min of exposure. The FEM simulations of the same locations predicted temperatures of 29.08 °C and 28.47 °C, respectively, which is a negligible deviation from the actual temperature change. Figure 12 displays the Multiphysics FEM simulation results of 3C-SiC and Pt on a Si substrate [85]. The simulated results indicated a noticeable difference in the induced heat and SAR, with 3C-SiC showing 30% less SAR than that of Pt. However, both samples had very low levels of SAR, most likely due to their small size. Larger electrodes, like the ones used in deep brain stimulators, would have considerably more heat generation. Overall, this study suggests that 3C-SiC may be an important part of an implantable neuromodulation device, providing low levels of image artifact and lower induced heat generation during exposure to high-energy MRI.

## 3. Discussion

Today’s chronically implanted neural implants have demonstrated many issues due to multiple biotic and abiotic factors. Many have demonstrated a glial inflammatory response that continues long after tissue damage from the implantation should have healed, while many have shown issues with corrosion, cracking, delamination, and electrical failure. While multiple explanations for these observations have been reported in the literature, we believe that a major cause stems from problems associated with the heterogeneous integration of dissimilar materials, such as silicon, metal electrodes, polymer insulation, etc. Silicon carbide is a material that is physically robust, chemically inert, and has multiple crystalline forms with different electrical properties. The amorphous form is insulating, while the crystalline form is semiconducting and can be doped to act either as an electron donor or acceptor. The amorphous form can be deposited at low temperatures using PECVD, enabling it to act as a protective coating over fabricated devices. It can also be used as both the insulator and the physical structure of a penetrating neural implant, thus creating an implantable device that is small but strong enough to be implanted without assistance. To support this assertion, *a*-SiC chronic neural implants have shown excellent biocompatibility and electrical reliability in vivo up to 16 weeks. As it can also withstand high temperatures, *a*-SiC neural interfaces can be constructed using pyrolyzed photoresist film to synthesize patternable carbon (C) traces to replace metals that have chronically shown corrosion issues in vivo.

A comparison of both the all-SiC and C-based SiC electrodes has been compiled by Saddow [59] and is shown below in Table 2. For comparison purposes, platinum (Pt), iridium oxide (IrO_x_), and titanium nitride (TiN) are also included. While direct comparison based on literature data is not always one-to-one, from this table, one can see that 3C-SiC and carbon electrodes sandwiched in the *a*-SiC matrix demonstrate a superior impedance and charge storage capacity compared to Pt, IrO_x_, and TiN, which is a very encouraging result.

Table 2 indicates that crystalline SiC has the capability to provide an excellent platform for recording, as it possesses impedances that are on par or even better than conventional metals and conductive polymers. Iridium oxide and titanium nitride, two materials that are incorporated into many different neural implants, show higher impedances, even with electrodes that have a smaller overall area. However, carbon derivatives, like graphene fibers and PPF, demonstrate lower overall impedances than SiC on average. This result is not surprising, as carbon, especially graphene, is a well-known conductive material, and in many ways, silicon carbide is a related material. It can be noted that these two materials can be incorporated easily into a heterogeneous neural implant device, as demonstrated with a-SiC/PPF neural implants in Section 2.2, thereby combining the physical resilience and chemical resistance of SiC with the electrochemical properties of carbon. While the table notes that SiC demonstrates excellent impedance and charge storage capacity, there is a notable difference that is not shown in the table. The electrochemical modeling of SiC provided in Section 2.3 indicates that it is a material that interacts with the electrochemical environment through capacitive means instead of Faradaic chemistry, like iridium oxide. This factor introduces limitations of electrical interaction when considering lower frequency transmissions, as the capacitive nature of SiC will attenuate these signals. Stimulation can also become a problem, as the electrodes would require more time to discharge than an equivalent Faradaic electrode.

If one uses crystalline SiC, a truly monolithic device can be created using a single material. One of the abiotic issues with present-day neural interfaces is that they require multiple materials to construct, namely insulating and conducting materials. Many of these materials have difficulty interacting together, often leading to material delamination. With crystalline SiC, the insulator and conductor can be replaced with a pn junction, which has been shown to provide excellent isolation between adjacent traces and prevent electrical leakage into the substrate. The electrode impedance has also been shown to be comparable to a metal electrode of the same size. Another benefit is the available water window, or the point where hydrolysis occurs, which has increased from 0.8 V to 2.7 V for platinum and 4H-SiC, respectively. Another advantage of using only SiC in neural implants is that it can reduce negative reactions within a high-power MRI magnetic field. Even at 7T, SiC has shown nearly no image artifacts, and it is predicted to have 30% less SAR than Pt metal conductors. Based on this reviewed and presented research, SiC has been demonstrated as a promising candidate material for improving the reliability of chronic neural implants.

## 4. Conclusions

Neural implants now play a critical role across modern medicine, enabling sensory and motor restoration, treating neurological and psychiatric disorders through targeted neuromodulation, and providing high-fidelity neural interfaces that support advanced bionic limb control. Together, these technologies demonstrate the broad and growing impact of implantable neural interfaces on human health and quality of life. However, most clinically deployed devices rely on macroelectrodes with areas on the order of mm^2^, which lack specificity and can exhibit deleterious effects when exposed to MRI protocols. Microelectrodes have shown that they can serve as effective neural implants, allowing for both stimulation and recording of specific neural tissue, yet they have not demonstrated the chronic reliability needed for long-term human use. The well-documented chronic reliability failures of neural implants have been attributed to numerous biotic and abiotic factors, many of which stem from the materials used in device fabrication.

Silicon has long been a mainstay material in the neural implant field due to its extensive processing infrastructure and compatibility with a variety of conductors and insulating materials. Unfortunately, the very material properties that make silicon an excellent fabrication platform have also contributed to the chronic failures observed in long-term implantation. Silicon carbide (SiC), a semiconductor material similar to silicon but with greater physical resilience and near-complete chemical inertness, offers a compelling alternative. Amorphous SiC (*a*-SiC) can be deposited via PECVD at temperatures compatible with polymer substrates, while crystalline SiC can be doped to achieve metallic-like conduction. SiC is compatible with established silicon semiconductor-manufacturing techniques, enabling precise fabrication of neural implants. Notably, *a*-SiC has been shown to improve device reliability in platforms such as the Blackrock Utah Array by protecting parylene insulation from the harsh neurochemical environment, and it allows for the fabrication of implants with very small cross-sections that can be inserted without shuttles, thereby reducing acute tissue damage and improving chronic reliability.

A major abiotic limitation of traditional neural implants is the interaction between the various heterogeneously integrated material layers. These interactions, coupled with the neural environment, frequently lead to delamination, cracking, electrical leakage, and other catastrophic failures. Crystalline SiC presents a potential solution through the use of pn junctions. By doping single-crystal SiC with electrically active dopants, a pn junction forms at the interface between p-type and n-type regions, creating a depletion layer that blocks current and enables a homogeneously integrated neural implant—a truly monolithic device composed of a single material system. In such devices, heavily doped n-type SiC serves as the conductive pathway on a p-type base layer, eliminating the need for metal traces or separate insulating materials to prevent leakage between adjacent conductors. Beyond simplifying the material stack, monolithic SiC demonstration devices have exhibited excellent impedance characteristics and charge storage capacity comparable to traditional metal electrodes.

One important consideration is that SiC’s semiconductor nature leads to predominantly capacitive electrochemical behavior, which can impact low-frequency signal transduction and complicate stimulation due to slower charge dissipation. Nevertheless, SiC offers significant advantages, including magnetic permeability nearly identical to neural tissue and thermal conductivity approaching that of copper at room temperature. These properties enable SiC-based implants to withstand high-resolution MRI fields of at least 7T without generating imaging artifacts. Thermal modeling further suggests that monolithic SiC neural implants should dissipate heat effectively across the device volume, reducing risks of localized heating. Overall, SiC has shown outstanding promise as a material capable of supporting chronically reliable neural implants suitable for long-term human therapeutic applications.

## Figures and Tables

**Table 1:** The impedance at 1 kHz of 900 °C PPF vs. Pt [59].

Electrode *	Area [kµm^2^]	Impedance 1 kHz [kΩ]	CSC_a_ [mC/cm^2^]	CSC_c_ [mC/cm^2^]
PPF	0.49	125.6 ± 25.7	5600	3000
Pt	0.49	437.8 ± 35.9	53.2	33.2
PPF	1.9	24.8 ± 0.4	8900	5260
Pt	1.9	103.8 ± 2.2	4.7	7.4
PPF	7.8	11.4 ± 0.04	2870	1600
Pt	7.8	51.8 ± 1.1	4.9	6.4
PPF	125	7.4 ± 0.04	193	107
Pt	125	8.4 ± 0.2	4.1	5.6
PPF	502	7.7 ± 0.01	50	27
Pt	502	6.3 ± 0.1	3.9	5.2

* Electrochemical window for PPF and Pt −0.7 V to 1.5 V and −0.6 V to 0.8 V, respectively.

**Table 2:** Electrochemical properties of SiC and common electrode materials [59].

Material	Recording Area [kµm^2^]	Impedance @ 1 kHz [kΩ]	Charge Storage Capacity [mC/cm^2^]
3C-SiC [65]	0.49	71.6	205
PPF/*a*-SiC [65]	1.9	24.8	14.160
Pt [65]	1.9	103.8	12
PEDOT/CNT [87]	2.83	15.0	6
Carbon-nanotube fiber [88]	1.450	14.1	372
Graphene Fiber [89]	0.749	37.9	798
IrO_x_ [90]	0.177	132.9	29
TiN [87]	2.83	54.8	5

## Data Availability

No new data were created or analyzed in this study.

## Abbreviations

The following abbreviations are used in this manuscript:

3C-SiC	3 Cubic—Silicon Carbide
4H-SiC	4 Hexagonal—Silicon Carbide
*a*-SiC	Amorphous Silicon Carbide
BCI	Brain–Computer Interface
BMI	Brain–Machine Interface
CSC	Charge Storage Capacity
CV	Cyclic Voltammetry
DRIE	Deep Reactive Ion Etching
EIS	Electrochemical Impedance Spectroscopy
FOV	Field of View
GSA	Geometric Surface Area
HMDS	Hexamethyldisilazane
IDE	Interdigitated Electrode
INI	Intracortical Neural Interface
LOR	Lift-off Resist
MEA	Microelectrode array
mINP	Microelectrode Implantable Neural Probe
MRI	Magnetic Resonance Imaging
PBS	Phosphate-Buffered Saline
PECVD	Plasma-enhanced Chemical Vapor Deposition
PPF	Pyrolyzed Photoresist Film
RARE	Rapid Acquisition with Relaxation Enhancement
RCA	Radio Corporation of America
RIE	Reactive Ion Etching
RTP	Rapid Thermal Processor
SiC	Silicon Carbide
SOI	Silicon on Insulator
TE	Echo time
TR	Repetition time
USF	University of South Florida
